# Checklist for Early Recognition and Treatment of Acute Illness (CERTAIN): evolution of a content management system for point-of-care clinical decision support

**DOI:** 10.1186/s12911-016-0367-3

**Published:** 2016-10-03

**Authors:** Amelia Barwise, Lisbeth Garcia-Arguello, Yue Dong, Manasi Hulyalkar, Marija Vukoja, Marcus J. Schultz, Neill K. J. Adhikari, Benjamin Bonneton, Oguz Kilickaya, Rahul Kashyap, Ognjen Gajic, Christopher N. Schmickl

**Affiliations:** 1 Multidisciplinary Epidemiology and Translational Research in Intensive Care (M.E.T.R.I.C.), Division of Pulmonary and Critical Care Medicine, Mayo Clinic, 200 1ST Street SW, Rochester, MN USA; 2 The Institute for Pulmonary Diseases of Vojvodina, Sremska Kamenica, Faculty of Medicine, University of Novi Sad, Novi Sad, Serbia; 3 Academisch Medisch Centrum, Universiteit van Amsterdam, Amsterdam, Netherlands; 4 Department of Critical Care Medicine, Sunnybrook Health Sciences Centre and University of Toronto, Toronto, Canada; 5 Emergency Department, René Arbeltier Hospital, Coulommiers, France; 6 Department of Medicine, Boston Medical Center, Boston University School of Medicine, Boston, MA USA; 7 Department of Anesthesiology and Reanimation, Gulhane Military Medical Faculty, 06010 Etlik, Amkara Turkey

**Keywords:** Point-of-care, Decision-support tool, Checklist, Content, Infrastructure, Critical care, Software, Technology

## Abstract

**Background:**

The Checklist for Early Recognition and Treatment of Acute Illness (CERTAIN) is an international collaborative project with the overall objective of standardizing the approach to the evaluation and treatment of critically ill patients world-wide, in accordance with best-practice principles. One of CERTAIN’s key features is clinical decision support providing point-of-care information about common acute illness syndromes, procedures, and medications in an index card format.

**Methods:**

This paper describes 1) the process of developing and validating the content for point-of-care decision support, and 2) the content management system that facilitates frequent peer-review and allows rapid updates of content across different platforms (CERTAIN software, mobile apps, pdf-booklet) and different languages.

**Results:**

Content was created based on survey results of acute care providers and validated using an open peer-review process. Over a 3 year period, CERTAIN content expanded to include 67 syndrome cards, 30 procedure cards, and 117 medication cards. 127 (59 %) cards have been peer-reviewed so far. Initially MS Word® and Dropbox® were used to create, store, and share content for peer-review. Recently Google Docs® was used to make the peer-review process more efficient. However, neither of these approaches met our security requirements nor has the capacity to instantly update the different CERTAIN platforms.

**Conclusion:**

Although we were able to successfully develop and validate a large inventory of clinical decision support cards in a short period of time, commercially available software solutions for content management are suboptimal. Novel custom solutions are necessary for efficient global point of care content system management.

**Electronic supplementary material:**

The online version of this article (doi:10.1186/s12911-016-0367-3) contains supplementary material, which is available to authorized users.

## Background

Checklists are a simple way to reduce errors in complex high-risk environments. Widely used in aeronautics for decades, Gawande et al. recently publicized the use of checklists in medicine [1, 2]. These observational studies demonstrated improvements in safety and outcomes when integrated into operating room routines, both in high-resource and low-resource countries [1, 3]. A simulation study by the same group further suggests that checklists also significantly improve surgical care during emergency situations where rapid and correct decision-making is crucial to ensure good patient outcomes [4].

Building upon these experiences and advances in informatics and human factor engineering, a novel electronic tool, the Checklist for Early Recognition and Treatment of Acute Illness and Injury (CERTAIN), is being developed by a large international collaboration with the overall objective of standardizing the approach world-wide to the evaluation and treatment of critically ill patients, in accordance with best-practice principles [5]. Similar to surgical checklists, CERTAIN may be particularly beneficial in low-resource settings with a scarcity of formally trained personnel [6].

**Fig. 1:**
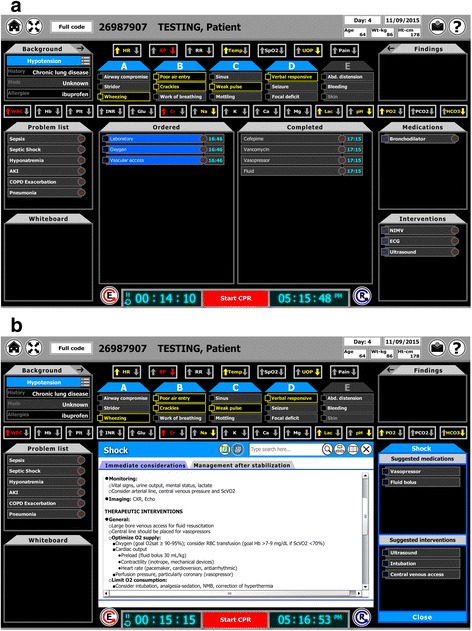
Panel **a** shows CERTAIN’s main display facilitating a structured approach to acutely decompensating patients. Panel **b** shows the integrated on-demand clinical decision support for the syndrome card “shock” in the center of the screen

During the evaluation of acutely decompensating patients, CERTAIN guides health-care providers through a structured approach, starting with a primary survey (ABCDE) followed by a secondary patient survey consisting of reason for admission, past medical history and the patient’s problem list. The latter is the gateway to the clinical decision support embedded into CERTAIN. In CERTAIN software, selection of a syndrome on the problem list leads to on-demand display of point-of-care key information in an index card format in the center of the screen with recommendations regarding further diagnostic and therapeutic steps (Fig. 1). Similarly, point-of-care key information is readily available or “just one click away” for selected procedures and medications. The process of creating and maintaining concise, accurate and up-to-date information in index card format (“cards”) for clinical decision support in CERTAIN is thus the cornerstone to CERTAIN’s success. Our experiences and lessons learned regarding this process will be the subject of the remainder of this paper (information about other aspects of the CERTAIN project including more analytical evaluations are available elsewhere [5, 7–9]).

## Methods

*A priori* we postulated that an ideal *content management system* for CERTAIN should have the following characteristics:

### Content

The content should cover a wide variety of clinically important topics, be easy to read, contain useful point of care information based on up-to-date evidence and be validated by expert reviewers. The content would initially be produced and deployed in English but translated into local languages as the project develops. The content should also be applicable in care environments which may have resource limitations and adaptable to local circumstances. The information provided should be supported by key references, web-links, and videos (e.g. demonstrating procedures) as appropriate. Given our objective to provide structured bedside decision support based on *existing* guidance and evidence, we deliberately decided against a process involving the development of *new* guidelines.

### Infrastructure

The data management system that supports the clinical content must restrict access to authorized personnel and allow frequent, automated back-ups. Information should be stored centrally with the capability to instantly update all the different CERTAIN platforms (software, mobile application, PDF-booklet available for download and print). The data management system must further provide ease of access for authors and reviewers to facilitate development and validation of the content.

## Results

We developed content for the software using the following steps. First, Bonneton et al. undertook an international survey of critical care professionals to identify the most common medical syndromes, medications, and procedures in acute care [7]. This survey also assessed the types of information considered to be most pertinent by bedside care providers (e.g. diagnostic tests vs epidemiologic data) [7]. Based on these survey results, we defined three different card categories – syndromes, medications, procedures – and created templates for each (for examples see Additional files 1, 2 and 3). A Mayo Clinic-based research physician, the “content lead editor”, organized the development and validation process for all cards (see Additional file 4: Figure S1 and Additional file 5: Figure S2).

### Creation

Specific cards are drafted by content matter experts utilizing primary literature and published guidelines identified via multiple databases (e.g. Medline, EMBASE, National Guideline Clearing House, Cochrane Library, Up-To-Date). Both authors and reviewers are selected from the “expert panel”, a convenience sample of (currently) 81 physicians and 6 pharmacists from various backgrounds (e.g. critical care, anesthesia, emergency medicine), including many physicians involved in an ongoing study implementing CERTAIN into clinical practice [5].

### Review and proofreading

Completed drafts of cards are then validated using an open peer-review process (Additional file 5: Figure S2): each new card is assigned to 2–3 reviewers chosen from the expert panel. Reviewers have the option to request reassignment to other cards based on their preference or expertise. Minor reviewer comments (e.g. grammar or spelling issues) are directly corrected by the content lead editor. Major comments are resolved by open discussion involving the original author, the other assigned reviewers, and the content lead editor who is supported by, and can seek further advice from, the “content management panel” at any time. This latter group consists of five senior physicians who are also part of the expert panel. Although all major comments to date have been resolved using this process, for content issues which remain unresolved, a mechanism exists to facilitate final arbitration via a modified Delphi process involving the entire expert panel [10].

### Publication and updates

After a card is proofed and finalized, it serves as the blueprint to update the different CERTAIN platforms. To ensure that the content stays up-to-date, authors receive a request after one year to update their card with regards to changes in current evidence and guidelines, followed by the same review process as when creating a new card. In addition, this updating process can be triggered at any time by any CERTAIN user via an embedded feedback button in the software. While the main work flow for development and validation is based on the English content, cards are currently being translated simultaneously to other languages including Spanish, Chinese, Turkish, Croatian, Serbian, and Polish. We currently have 60 Spanish, 176 Chinese,152 Turkish,20 Serbo-Croatian and 65 Polish cards. The process is similar to the general validation process: experienced bilingual clinicians translate the cards which are then reviewed by bilingual peers prior to incorporation into the various CERTAIN platforms (for more details see Additional file 6).

### Ownership

The coordinating center overseeing content management across all platforms is the Mayo Clinic, Rochester. This role includes development, review, updates, and translation of content and infrastructure and involves system organization, reminding authors and reviewers about update deadlines, recruiting authors, reviewers and translators for new content development and working with programmers to find technical solutions for data management issues. Individual card “ownership” and authorship is shared between the author and reviewers and acknowledged on all CERTAIN output (software, mobile app, PDF). If any original authors/reviewers cannot maintain “ownership” of a card the coordinating center assigns new individuals to adopt that card.

## Discussion

### Card authorship

While the general process outlined above has remained constant over time, many practical details were refined due to changing circumstances and needs as summarized in Table 1. For example, one major change was the increase in the number of authors (See-Appendix 6 list of authors/reviewers). Initially, these were restricted to a few members of our research group to facilitate rapid growth and consistent standards of the card inventory. However, given the need for annual revisions, it has not been feasible for one author to take ownership of more than just a few cards. The current number of cards created by 36 authors includes 67 syndromes, 117 medications, and 30 procedures (for a full list of cards see Appendix 5). Thus, over time we recruited more collaborators, often colleagues of original authors and reviewers, and trainees at the fellow level in the pulmonary and critical care department at our institution. So far 127 (59 %) cards have been reviewed, with each reviewer being assigned an average of two cards (range 1–7 cards).

**Table 1:**
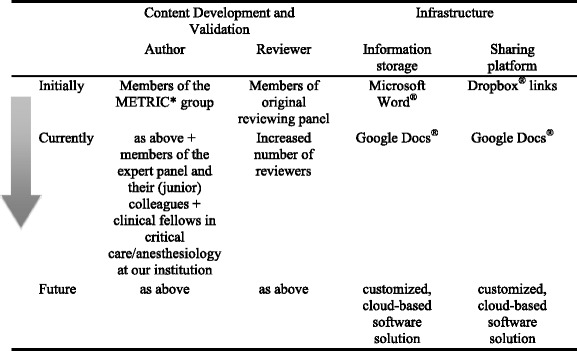
Summary of the evolution of the content management system

^a^ *METRIC* Multidisciplinary Epidemiology and Translational Research in Intensive Care

### Software infrastructure

Cards were initially created, stored and modified in Microsoft Word® using Dropbox® links for sharing. Using those links, reviewers could download the Word® files, revise and add comments to the documents and then send them back to the content lead editor as an email attachment. Unfortunately Dropbox® did not support real time collaboration between different authors simultaneously. While technologically simple, this method became impractical due to the large amount of time needed to organize the collaboration process to enable reviewers to see each others’ comments and hence facilitate a virtual discussion. The potential for missed emails and slow file updates, and the lack of version control became unworkable for the content lead editor. We improved this process by switching to Google Docs®, which allows sharing of text documents via links and editing of the source files directly by multiple reviewers simultaneously. Drawbacks of this approach included loss of much of the initial formatting and the inability to easily create and update a PDF booklet. Microsoft Word® allowed linkage of all single documents together in one master file with an automated table of contents, creating a booklet which could be updated within minutes. In addition, although freely available in general, access to Google Docs® is restricted in certain countries (e.g. China) where some of our authors and reviewers live, thus limiting some collaborative opportunities.

Although not directly applicable to our project, because the various open source content management systems reviewed by Mooney et al. are geared towards creators of wikis or blogs, we agree with their final conclusion that when choosing an appropriate tool “first and foremost, security should be evaluated as well as the aptitude, availability and coverage of the user support community” [11]. Neither Google docs® nor Dropbox® met our security needs, to which there are several aspects: We want to be certain that cards can only be edited by invited and qualified individuals to ensure high quality of the cards which potentially impact clinical decision making and thus health outcomes; additionally we also want to enable local clusters (e.g. a specific hospital) or users to customize the content to reflect local/individual preferences with those modifications being editable and visible only by that local cluster or user, respectively; lastly the data needs to be backed up on *local* servers automatically at frequent intervals to ensure restorability and offline work in case of disrupted internet connection and/or failure of the cloud server etc. Of note, with regards to our content management system we are not concerned about any patient confidentiality issues, because while personal data can be entered into CERTAIN software (to facilitate charting and debriefing) the cards themselves do not contain any patient information.

Furthermore both Google docs® and Dropbox® required excessive work to execute simple tasks, including *manually* updating the software and mobile applications whenever content changed; sending email reminders to reviewers to ensure compliance with peer review deadlines; and keeping track of the cards’ expiration dates. Due to the absence of readily available software meeting our requirements, we have thus started developing a customized software solution that will allow authors and reviewers to co-create, edit and review content directly in a secure cloud server with the capability to *automatically* update the different platforms including the PDF booklet (see Table 2 and Additional file 7: Figure S3).

**Table 2:** Infrastructure of the customized content management system

Infrastructure services from AWS	Function
EC2	EC2 and Amazon Machine Image used to create the virtual machine. Then Apache Server and PHP programming environment inside each instance were installed as our web/app server environment.
S3 Storage	Used for saving our application development files
CloudFront	Used CloudFront as a Content Delivery Network (CDN) in order to provide a contents distribution to end users with low latency, high data transfer speeds.
Mongo lab	This document oriented database service on top of AWS EC2 provided the persistence layer for our contents storage and back-up service
VPC	Put our EC2 servers and RDS database into the VPC group in order to provide a more secured and isolated private network for all our cloud services.
IAM and Trusted Advisor	Security is always a top priority for a clinical study related application. By adopting these 2 services, we can create a secured strategy to enables us to securely control access to AWS services and resources for our users. Using IAM, we can create and manage AWS users and groups, and use permissions to allow and deny their access to our CERTAIN CMS application resources
Elastic load balancer	This AWS on-demand scaling load balancer and monitor system assured our application can be elastically expanded to support global usage in a most efficient way.
Software components:	
CERTAIN CMS web admin	HTML5 based web admin portal manages all the medical cards
CMS contents APIs	The web service server to be used by different CERTAIN client software (flash, CMS admin and mobile app)
MongoDB	MongoDB is the persistence layer for our content management system

In order to build this scalable applications platform for our study, we selected Amazon Web Service (AWS) as our Infrastructure as a Service (IAAS) provider. AWS is a global leader in this area and right now it provides more than 40 cloud services for its 11 geographical regions across the world for IT developers. After making an assessment of the quality, security risk, time and cost factors, we used the following infrastructure services from AWS to build our CERTAIN CMS platform

While we have been somewhat struggling to find the right infrastructure, we have made much progress in the development and validation of the content itself, despite essentially no funding. Within three years we were able to create more than 200 cards, with more than half of them being validated. In large part this was only possible by directly involving many of the CERTAIN end-users into the content-creation process, thus reducing the work load per person while increasing the users’ “buy-in” into the CERTAIN concept.

While our approach of an open peer-review may increase the risk of “herding” (ie. reviewers being more likely to be influenced by and agreeing with their peers’ potentially incorrect opinions), [12] we intentionally adopted this process because open peer-review is generally thought to increase accountability, fairness, and transparency, with some evidence showing that it leads to better quality reviews, while being preferred by authors [13–16]. Despite the theoretical risk that identifiable reviewers may feel more hesitant to criticize their peers, in practice the loss of anonymity does not appear to significantly affect reviewers’ decision to ask for major revisions or reject manuscripts [15]. While “open peer review” generally just denotes that reviewers’ identity is revealed to the authors (and possibly to the readers if the manuscript under review is eventually accepted) we further enabled reviewers to be aware of each other’s identity and opinions as well. This may further increase the risk of “herding”, but at a time where medical knowledge doubles approximately every 3 to 4 years we feel it is best to have an open and maximally transparent discourse involving as many content experts as possible [17]. This principle of maximal inclusion of content experts also underlies the mechanism to resolve complex issues using a modified Delphi process involving the *entire* expert panel and our efforts to encourage each CERTAIN user to simultaneously function as a peer-reviewer via the feedback option within the software.

A crucial challenge in developing *globally* applicable decision support is to provide best-practice recommendations that are relevant in low-resource practice settings where some tests and interventions may not be available. We tried to solve this conundrum by crafting cards based on best evidence assuming a resource-rich setting while allowing users to add permanent contextualizing card notes to each card. In the future we further plan to add this feature for clusters, so that for instance hospitals can add a specific recommendation regarding antibiotics for pneumonia taking into account local resistance patterns and drug availability, which will be visible to all providers affiliated with that particular hospital or cluster.

There have been many other attempts to harness the technologic progress to improve and standardize health-care in under-developed and under-served regions: for example, within the United States about 6 % of all intensive care patients are now (co-)managed by a telemedicine intensivist who remotely monitors patients’ condition in real-time and provides support to the onsite personnel [18]. This remote expertise and standardization of care appears to improve outcomes, but major barriers include the need and cost for 24/7 available remote experts, variable acceptance by the onsite personnel (who may feel monitored rather than supported) as well as variable integration and interoperability with the local software environment [18]. In an interesting extension of the telemedicine concept to resource-poor countries Celi et al. realized that a major infrastructure asset is the rampant availability of mobile phones which allow access to and interchange of information even in settings devoid of wireless internet and computers, and thus created a “cell phone-facilitated clinical system” [19]. This system, which is integrated into open MRS (an open source medical records system), allows users (e.g. patients or local health workers) to send medical information including images and voice messages to remote specialists who in turn could provide live decision support. While we similarly encountered software issues described above, CERTAIN alleviates the acceptance issue by providing *on-demand*, interactive best practice advise to the onsite providers, empowering them to use, ignore or modify the information at their own discretion. Additionally, and similar to the project by Celi et al., CERTAIN tries to address the challenge of getting technologic support to areas with potentially minimal infrastructure and/or internet capability by offering decision support via various platforms including a paper version as well as a mobile phone app.

Another major challenge at the intersection between technology and healthcare that we encountered with CERTAIN itself is the difficulty of keeping a balance between doing justice to the complex environment that it is being designed for (i.e. evaluation of critically ill patients) while keeping the interface simple since in these situations time is generally of the essence. For example, during a recent simulation study participants largely provided positive feedback about CERTAIN in general, but felt that the software should become somewhat more intuitive. It is reassuring though, that in this simulation study CERTAIN improved health-care providers’ performance [20]. It is unclear, however, whether the benefit is due to its embedded decision support or the other components such as teaching a structured approach to patient care, safety culture, and closed loop communication strategies. A before-after quality improvement study is underway to assess the impact of CERTAIN on care processes and patient outcomes when implemented into clinical practice in multiple Intensive Care Units (ICUs) across five continents after training local personnel remotely via live video stream [5, 9]. This study will significantly increase the number of users who can provide feedback about how well the decision support aligns with frontline providers’ needs. Based on this feedback, we are continuously improving the decision support system with a special focus on workflow integration, data entry and output, standards and transferability, and knowledge maintenance [21]. However, if shown to improve processes of care or patients’ outcomes, future research will be needed to determine the relative contribution of CERTAIN’s different components.

## Conclusions

Although we were able to successfully develop and validate a large inventory of clinical decision support cards in a short period of time, readily available software products are suboptimal for use as content management platforms, requiring us to pursue a customized software solution.

## E-Appendix 5 List of current cards Syndrome Cards

### Syndrome Cards

Abdominal Compartment Syndrome

Hypoglycemia

Acid base disorders-Metabolic acidosis

Infective Endocarditis

Acid base disorders-Metabolic alkalosis

Intoxication-Aspirin Overdose

Acid base disorders-Respiratory Acidosis

Intoxication-Benzodiazepines overdose

Acid base disorders-Respiratory Alkalosis

Intoxication-Beta Blockers overdose

Acute Abdomen

Intoxication-Calcium Channel Overdose

Acute Asthma

Intoxication-Digoxin Overdose

Acute Coronary Syndrome

Intoxication-Ethylene Glycol

Acute Kidney Injury

Intoxication-Opioids overdose

Acute Neuromuscular Disorder

Intoxication-Tylenol Overdose

Acute Pancreatitis

Liver Failure

Acute Pericarditis-Tamponade

Malaria

Acute Respiratory Distress Syndrome

Meningitis

Alcohol withdrawal

MERS

Anaphylaxis

Pain

Anxiety

Pleural Effusion

Aortic Dissection-Aortic Aneurysm Rupture

Pneumonia

Aspiration Pneumonia-Pneumonitis

Pneumothorax

Atelectasis

Pulmonary Embolism

Bradyarrhythmia

Sepsis

Burn

Severe Hypertension

C. Difficilis Colitis

Shock

Cardiogenic Pulmonary Edema

Spinal Cord Injury

Coma

Status Epilepticus

COPD Exacerbation

Stroke

Delirium

Tachyarrhythmia

Diabetic Ketoacidosis

Traumatic Brain Injury

Electrolyte imb-Hypercalcemia

Upper Airway Obstruction

Electrolyte imb-Hyperkalemia

Electrolyte imb-Hypermagnesemia

Electrolyte imb-Hypernatremia

Electrolyte imb-Hypocalcemia

Electrolyte imb-Hypokalemia

Electrolyte imb-hypomagnesemia

Electrolyte imb-Hyponatremia

Filoviral infections: Ebola

Gastrointestinal Bleeding

Hematuria

Hemoptysis

Hypercapnic Respiratory Failure

Medication Cards

Acetaminophen

Esmolol

Morphine

Acetylcysteine

Etomidate

Naloxone

Acid Blockers

Famotidine

Naltrexone

Acyclovir

Fentanyl

Neuromuscular blocking agents

Albumin

Fluconazole

Nicardipine

Albuterol

Flucytosine

NimodipineAlteplase

Fluid

Nitroglycerin

Amikacin

Flumazenil

Norepinephrine

Aminocaproic Acid

Furosemide

Octreotide

Amiodarone

Gentamicin

Omeprazole

Amlodipine

Glucose

Ondansetron

Amphotericin B Conventional

Haloperidol

Opioids

Amphotericin B liposomal

Heparin

Oxycodone

Ampicillin

HIV-Delavirdine

Oxymorphone

Ampicillin sulbactam

HIV-Dolutegravir

Pantoprazole

Anti-Histamines

HIV-Efavirenz

Phenytoin

Antimicrobial Therapy

HIV-Elvitegravir

Piperacillin-Tazobactam

Aspirin

HIV-Enfuvirtide

Potassium chloride

Atracurium

HIV-Etravirine

Propofol

Atropine

HIV-Maraviroc

Ranitidine

Azithromycin

HIV-Raltegravir

Reteplase

Benzodiazepine

Hydrocortisone

Rocuronium

Beta blocker

Hydroxyethyl starch

SMX-TMP

Bronchodilator

Ibuprofen

Sodium bicarbonate

Ca channel blocker

Imipenem

Statins

Calcium

Immunoglobulins

Steroids

Caspofungin

Insulin

Streptokinase

Cefepime

Intralipid

Succinylcholine

Ceftriaxone

Ipratropium

Tenecteplase

Ciprofloxacin

Ketamine

Terlipressin

Cisatracurium

Levofloxacin

Theophylline

Clindamycin

Lorazepam

Thiamine

Dexamethasone

Magnesium sulfate

Thrombolytics

Dexmedetomidine

Mannitol

Tramadol

Diazepam

Meropenem

Vancomycin

Digoxin

Metoclopramide

Vasopressin

Diltiazem

Metoprolol

Vasopressors

Diphenhydramine

Metronidazole

Vecuronium

Dobutamine

Midazolam

Verapamil

Epinephrine

Milrinone

Procedure Cards

Cardioversion

Chest tube

CT

Cultures

ECG

Family meeting

Flexible Bronchoscopy

Head Of Bed Elevation

Hemostasis

Informed consent and shared decision making

Endotracheal Intubation

Invasive Mechanical Ventilation

Laboratory

Lumbar puncture

MRI

NG/OG tube (suction)

Non-Invasive Mechanical Ventilation

Oxygen

Palliative care

Pericardiocentesis

Source control

Therapeutic Hypothermia

Transfusion (type screen)

Transcutaneous pacing

Ultrasound

Urinary catheter

Vascular access (central line)

X-ray

Hemodialysis

Thoracocentesis

## Appendix 6

### Authors and Reviewers

**Table:**

A. Akhoundi	AaronTande
Abhay Vakil	Adil Ahmed
Alexander Kogan	Alenjandro Rabinstein
Alice Gallo de Moraes	Arthur Kwitzera
Guillermo Dominguez	Behj Riviello
Amra Sakusic	Benjamin Dreesman
Andrew Prince	Bhanu Gupta
Stacey A. Rizza	Bradley Peters
Carlos Racedo Africano	Brian Erstad
Chaitanya Undavalli	Brian Pickering
Rashid Ali	Daryl J Kor
Devang Sanghavi	David Kaufman
Essien Ekong	Deepak Kumar
Gustavo Matute-Bello	Emil J Manzur
Gina Lacovella	Emir Festic
Gwen Thompson	Enrique Ortiz-Diaz
Habtamu Belete	Erin Frazee
Harsheen Kaur	Garette Schramm
Humberto Sasieta Tello	Gavin Divertie
John C O’Horo	Gilsoul Hernandez Thierry
Joseph Zachariah	Gina Locovella
Kumar Sarvottam	Myung Park
Mustafa Arslantas	Michelle Biehl
Michelle Gong	William Atchley
Michael E. Wilson	Hasan Aldorzi
Muhammad Rishi	Heather Personette
Mustafa Arslantas	Hiroshi Sekiguchi
Perliveh Carrera	Jakobus Preller
Pramod Guru	Janice Tsui
Pujan Kandel	Jeffrey Rabatin
Markos Kashiouris	Jill Cherry-Bukoweic
Reddappa Kanipakam	John Dillon
Ronaldo Sevilla	John M Litell
Rudy Tedja	John Park
Srdjan Gajic	Jose Yunen
Sandhya Samavedam	Joseph Farmer
Saraschandra Vallabhajosyula	Jovan Matijasevic
Srdjan Gavrilovic	Kathleen To
Taru Dutt	Kelly Cawcutt
Venkatesh Gondhi	Kianoush Kashani
Vimal Ravi	Linda Bucher
Martin Duenser	Maja Surbatovic
Ohoud Aljuhani	Pedja Kovacevic
Yaseen Arabi	Venu Velagapudi
Pablo Moreno-Franco	Jack O'Horo
Tyler Albert	Pauline Park
Phillippe Bauer	Pritish Tosh
Rafael Tolentino	Ronaldo Sevilla
Rajyabardhan Pattnaik	Sandeep Tripathi
Richard Hinds	Sanjay Subramanian
Raj Dhokarh	Sarah Hocker
Rob Fowler	Sarah Lee
Rodrigo Cartin-Ceba	Teng Moua

## Additional files

Additional file 1: Example of Syndrome Card. (DOCX 21 kb) Additional file 2: Example of Medication Card. (DOCX 19 kb) Additional file 3: Example of Procedure Card. (DOCX 19 kb) Additional file 4: Figure S1. Card Production Process. After cards are created they undergo iterative cycles of review and content modification. Once finalized and proofed, they are published across the different CERTAIN platforms. Updates, starting the process essentially from the beginning, are performed annually and on an as needed basis. (PDF 181 kb) Additional file 5: Figure S2. Peer-review work flow. For creation of a new card, card expiration and review, or user comments via CERTAIN’s feedback function, a peer review process is initiated. Most issues are resolved on discussion with the author, the content lead editor and the reviewers. However, for complex issues the content management panel can be involved, and if still unclear, final arbitration can be obtained using a modified Delphi process involving the entire expert panel. (PDF 147 kb) Additional file 6: Translation Process. (DOCX 424 kb) Additional file 7: Figure S3. Overview of customized content management system (for details see Table 2). (PDF 68 kb)

## Abbreviations

ABCDE: Airway, breathing, circulation, disability, exposure
CERTAIN: Checklist for early recognition and treatment of acute illness
Google docs: Google documents
ICU: Intensive care unit
Mobile apps: Mobile applications
MS Word: Microsoft word
pdf: Portable document format
