# Supplementary material for: Checklist for Early Recognition and Treatment of Acute Illness (CERTAIN): evolution of a content management system for point-of-care clinical decision support
Source: BMC Med Inform Decis Mak. 2016 Oct 3;16:127. doi: 10.1186/s12911-016-0367-3 (PMC5048402; doi:10.1186/s12911-016-0367-3)
Supplement: Additional file 1: — Example of Syndrome Card. (DOCX 21 kb) [file 12911_2016_367_MOESM1_ESM.docx]

**E-APPENDIX 1.** Example of a syndrome card

## GASTROINTESTINAL BLEEDING (GIB)

*(Last updated 06/08/2015; Author: Benjamin Bonneton, MD; Reviewers: Martin Duenser, MD; Neill Adhikari, MD)*

**1) IMMEDIATE CONSIDERATIONS**

**FINDINGS / DIAGNOSTIC INTERVENTIONS**

- **Upper GIB:**
  - **History:** cirrhosis, alcohol abuse, malignancy, aspirin/NSAIDs use, repeated vomiting (Mallory-Weiss tear), radiation
  - **Physical Findings:** check vital signs for evidence of shock(pulse/heart rate/BP/peripheries), hematemesis (red blood/hematin/coffee ground vomiting), hematochezia, melena, abdominal pain
  - **Consider nasogastric tube and diagnostic lavage(rarely done)**
  - **Underlying Causes:** Esophageal causes- esophageal varices/lesions, esophageal tear, esophagitis

Gastric causes-erosive gastritis or peptic ulcer disease, portal hypertensive gastropathy, gastric tumors, gastric vascular abnormalities, aortoenteric fistulas,

- **Lower GIB:**
  - **History:** malignancy, NSAID use, diarrhea or constipation, abdominal or rectal pain, weight loss
  - **Physical Findings:** melena (rectal examination!), hematochezia. abdominal pain or mass
  - **Underlying Causes:** GI tumors, vascular abnormalities/lesions, inflammatory bowel disease, diverticulosis, colitis, hemorrhoids, rectal varices
- **Labs:** complete blood count, coagulation (INR/PTT), blood type, crossmatch and LFTs
- **Monitoring:** hemodynamic and respiratory status
- **Imaging Studies:** endoscopy (esophagogastroduodenoscopy, colonoscopy), computer tomography angiography or digital subtraction angiography (lower GIB)

**THERAPEUTIC INTERVENTIONS**

- **General:**
  - **Resuscitation** and early consultation of gastroenterology or general surgery for patients in shock
  - **Transfusion**
    - Correct anemia, (consider RBC transfusion for Hb < 7g/dL)
    - Correct thrombocytopenia (platelets < 50,000)
    - Correct any coagulopathy (INR >1.5, PTT >50 sec)
  - Consider **intubation** if aspiration risk, respiratory failure, severe hemodynamic instability, or borderline respiratory or hemodynamic status and planned endoscopy
- **Medications**:
  - Peptic ulcer: high dose proton pump inhibitor (omeprazole or pantoprazole) IV
  - Varices: vasoactive drug therapy (terlipressin or octreotide/somatostatin) IV
  - Antibiotic prophylaxis in any patient with cirrhosis and GI hemorrhage:
    - N[orfloxacin](http://www.uptodate.com/contents/norfloxacin-drug-information?source=see_link) or [ciprofloxacin](http://www.uptodate.com/contents/ciprofloxacin-drug-information?source=see_link)
    - IV [ceftriaxone](http://www.uptodate.com/contents/ceftriaxone-drug-information?source=see_link) in patients with advanced cirrhosis
- **Early endoscopic intervention (<24h):**
  - UGIB: Consider prokinetic agents (erythromycin/metoclopramide) if suspected fresh blood or clot in the stomach, in patients who are likely to have a large amount of blood in their stomach, such as those with severe bleeding. (30-90 minutes prior to endoscopy)
  - Clipping/injection/cautery/band ligation therapy +/- combination
  - **If endoscopic hemostasis not successful or feasible:**
    - LGIB: angiography to localize bleeding
    - Esophageal varices: balloon tamponade ( Sengstaken-Blackmore/Minnesota tubes) and/or transjugular intrahepatic portosystemic (TIPS) stent shunts
    - UGIB: Consider emergent interventional radiology (embolization) or surgery

**2) MANAGEMENT AFTER STABILIZATION**

- **Further Labs:** repeat blood count and coagulation testing, blood gas including lactate, consider Helicobacter pylori testing in patients with erosive gastritis or peptic ulcer disease
- **Monitoring:** hemodynamic and respiratory status, urine output: resolution of shock!
- **Further Interventions/Treatments:**
  - R**epeat endoscopic therapy** if needed
  - **Peptic ulcer:** high-dose intravenous proton pump inhibitor during 72h, consider empirical eradication therapy for suspected Helicobacter pylori infection (e.g. in duodenal ulcer disease)
  - **Varices:** vasoactive drug therapy (terlipressin or octreotide/somatostatin) for 3-5 days
  - **If cirrhosis:** prophylactic antibiotics and lactulose (via nasogastric tube and/or enema).
  - **Control body temperature, coagulopathies and thrombocytopenia** after resuscitation/transfusion
    - **Restrictive red blood cell transfusion (hemoglobin >7 g/dL or lower in younger subjects)** after shock resolution or if no shock

**3) REFERENCES & ACKNOWLEDGMENTS**

-Scottish Intercollegiate Guidelines Network (SIGN). Management of acute upper and lower gastrointestinal bleeding. A national clinical guideline. Edinburgh (Scotland): Scottish Intercollegiate Guidelines Network (SIGN); 2008 Sep. 57 p.

-Tham TC, James C, Kelly M. Predicting outcome of acute non-variceal upper gastrointestinal haemorrhage without endoscopy using the clinical Rockall Score. Postgrad Med J. 2006 Nov;82(973):757-9

-Rockall TA, Logan RF, Devlin HB, Northfield TC. Risk assessment after acute upper gastrointestinal haemorrhage. Gut. 1996 Mar;38(3):316-21.

-Sreedharan A, Martin J, Leontiadis GI, et al. Proton pump inhibitor treatment initiated prior to endoscopic diagnosis in upper gastrointestinal bleeding. Cochrane Database Syst Rev 2010;

-[The Role of Endoscopy in the Management of Acute Non-Variceal Upper GI Bleeding](http://www.asge.org/WorkArea/linkit.aspx?LinkIdentifier=id&ItemID=15154) Gastrointest Endosc 2012:75:1132-1138

-Transfusion Strategies for Acute Upper Gastrointestinal Bleeding. Villanueva C. N Engl J Med 2013; 368:11-21 [January 3, 2013](http://www.nejm.org/toc/nejm/368/1/)

-Prevention and management of gastroesophageal varices and variceal hemorrhage in cirrhosis. Garcia-Tsao G. Hepatology 2007; 46:922.

- [Villanueva C, Colomo A, Bosch A, et al. Transfusion strategies for acute upper gastrointestinal bleeding. N Engl J Med 2013; 368:11.](http://www.uptodate.com/contents/indications-and-hemoglobin-thresholds-for-red-blood-cell-transfusion-in-the-adult/abstract/42)

- [Garcia-Tsao G, Sanyal AJ, Grace ND, et al. Prevention and management of gastroesophageal varices and variceal hemorrhage in cirrhosis. Hepatology 2007; 46:922.](http://www.uptodate.com/contents/general-principles-of-the-management-of-variceal-hemorrhage/abstract/44)
