# Supplementary material for: Checklist for Early Recognition and Treatment of Acute Illness (CERTAIN): evolution of a content management system for point-of-care clinical decision support
Source: BMC Med Inform Decis Mak. 2016 Oct 3;16:127. doi: 10.1186/s12911-016-0367-3 (PMC5048402; doi:10.1186/s12911-016-0367-3)
Supplement: Additional file 2: — Example of Medication Card. (DOCX 19 kb) [file 12911_2016_367_MOESM2_ESM.docx]

**E-APPENDIX 2.** Example of a medication card

## OMEPRAZOLE

*(Last update: 9/28/2015; Author: Sandhya Samavedam, MD; Reviewers: Brian Erstad, Pharm D)*

**1) INDICATIONS**

**ADULTS**

- **Acute Gastrointestinal bleeding from peptic ulcer disease:**
  - **IV:** an initial bolus of 80 mg followed by 8 mg/hr infusion for the first 72 hours, followed by oral administration of omeprazole. IV FORM NOT AVAILABLE IN USA
  - **ORAL**: see below
- **Duodenal Ulcer, GERD:**
  - **ORAL:** 20 mg daily for 4-8 weeks.
- **Gastric Ulcer, Barrett's Esophagus:**
  - **ORAL:** 40 mg daily for 4-8 weeks.
- **Erosive Esophagitis:**
  - **ORAL:** Initial: 20 mg daily for 4-8 weeks followed by 20 mg/day for a year
- **Stress Ulcer Prophylaxis**
  - **ORAL:** 20 mg or 40 mg BID daily
- **H. Pylori:**
  - **ORAL:** 40 mg daily for 14 days along with other drugs

**PEDIATRICS**

- **Erosive Esophagitis:**
  - **ORAL:** 0.5 to 3.5mg/kg/day
- **GERD:**
  - **ORAL:** 0.7mg/kg/day for infants, 5 mg orally daily for children between 1-2 years; 10-20mg daily for 2 and above
- **Peptic Ulcer:**
  - **ORAL:** same as erosive esophagitis
- **H. Pylori eradication:**
  - **ORAL:**
    - Children: 1-2mg/kg/day, maximum 20mg daily
- **Hypersecretion of acid:**
  - **ORAL:** 0.7-2.75mg/kg/day

**2) CAUTIONS**

- **Main side effects:**
  - Osteopenia related bone fractures
  - Atrophic Gastritis
  - Clostridium Difficile
  - Interstitial Nephritis
  - B12 deficiency
  - Diarrhea and constipation with long term use among children
- **Dose adjustment:**
  - Renal problems: None
  - Hepatic insufficiency: May need dose adjustments

**3) AVAILABLE FORMS/ROUTES**

- **ORAL:**
  - Tablet or capsule 10 mg, 20 mg or 40 mg
  - Oral Suspension: 90 mL, 150 mL, 300 mL
- **IV:** infusion bag

**4) ORDER ENTRY (COMMONLY USED DOSES)**

- **ORAL:** 20 mg, 40 mg

**5) REFERENCES**

-AstraZeneca Pharmaceuticals. Prilosec Prescribing Information Website.<http://www1.astrazeneca-us.com/pi/Prilosec.pdf>. March, 2014. Accessed June 7^th^, 2015.

-Proton Pump Inhibitors. Use in Adults website.<http://www.cms.gov/Medicare-Medicaid-Coordination/Fraud-Prevention/Medicaid-Integrity-Education/Pharmacy-Education-Materials/Downloads/ppi-adult-factsheet.pdf>. August, 2013. Accessed June 7^th^, 2015.

-Israel DM., Hassall, E. Omeprazole and Other Proton Pump Inhibitors: Pharmacology, Efficacy, and Safety, with Special Reference to Use in Children. *J pediatr gastroenterol nutr*, 1998, 27(5): 568-579

-Fuccio L, Minardi M et al. [Meta-analysis: duration of first-line proton-pump inhibitor–based triple therapy for Helicobacter pylori eradication.](http://h)  *Ann of int med*. 2007. 147(8): 553-562.

-Zimmermann AE, Walters JK, Katona BG, et al. A Review of Omeprazole Use in the Treatment of Acid-Related Disorders in Children. *Clin Ther*, 2001, 23(5): 660-79.

-Lightdale JR, Gremse DA. Gastroesophageal Reflux: Management Guidance for the Pediatrician. *Pediatrics*, 2013, 131(5):e1684-95.
