# Supplementary material for: Checklist for Early Recognition and Treatment of Acute Illness (CERTAIN): evolution of a content management system for point-of-care clinical decision support
Source: BMC Med Inform Decis Mak. 2016 Oct 3;16:127. doi: 10.1186/s12911-016-0367-3 (PMC5048402; doi:10.1186/s12911-016-0367-3)
Supplement: Additional file 3: — Example of Procedure Card. (DOCX 19 kb) [file 12911_2016_367_MOESM3_ESM.docx]

**E-APPENDIX 3.** Example of a procedure card

## FLEXIBLE BRONCHOSCOPY

*(Last updated 04/27/2015; Author:  Humberto C. Sasieta Tello, M.D; Reviewers: Teng Moua, MD; Michelle Biehl, MD; Kelly Cawcutt, MD)*

**1) INDICATIONS**

**ADULTS**

- Diagnostic: Obtain washings, bronchoalveolar lavages, brushings and biopsies for histological, cytological and microbiological studies. Evaluate airway anatomy, hemoptysis origin.
- Therapeutic: Removal of foreign bodies, thick secretions, or mucous plugs. Fiberoptic intubation and endotracheal tube positioning

**PEDIATRICS**

- [same as adults]

**2) PROCEDURE**

- **Technical description:**
  - Flexible video bronchoscopy is the preferred modality, however direct vision bronchoscopes can also be used satisfactorily. Familiarity with the available equipment is very important. This procedure should be performed only by qualified and proficient providers.
  - Correct identification of patient, procedure, laterality and informed consent.
  - The patient must be placed on cardiac and pulse oximetry monitors, give 2-6 L/min of O_2_ by nasal cannula, and establish an IV access.
  - The use of a bite block, even in intubated patients is strongly recommended if bronchoscopy is performed through the mouth.
  - The mucosal surfaces and vocal cords are anesthetized with 1 cc squirts of Lidocaine 1% delivered through the bronchoscope’s working channel followed by flush of air to clear the channel. Pay special attention to the carinas. Any mucosal abnormality should be documented, indicating location and images.
  - Use moderate to general anesthesia depending on the duration of the procedure with short acting benzodiazepines like midazolam and opioids like fentanyl
  - After appropriate anesthetic of nasal (if performing via the nasal route) and oropharynx, gently insert nasally the flexible bronchoscope, an alternative option is orally
  - Once the vocal cords are visualized, inspect them for lesion and movement
  - To facilitate passage of the bronchoscope through the larynx, spray 1-2 ml of Lidocaine 1-2% through the working channel of the bronchoscope.
  - If the patient is awake ask him/her to take a deep breath to let the distal tip of the bronchoscope pass into the trachea, do not force through the vocal cords
  - Do a visual examination of the trachea, mainstem bronchi, and segmental bronchi
  - The removal of secretions can be obtained by simple aspiration or be aided with normal saline instillation. “Tapping” of the suction trigger is discouraged due to being ineffective. Suction is safe if no airway collapse, mucosal injury or hypoxemia develop.
- Bronchoalveolar lavage:
  - In diffuse conditions prefer right middle lobe or lingula. In focal conditions direct the bronchoscope to the most abnormal segment. Returns from lower lobes and posterior segments are more difficult and may require more aliquots.
  - Advance the bronchoscope to until wedged. Instill aliquots of saline in 20cc increments until distention of the airway is seen. Aspiration either by sequential suction through a trap, or syringe suction through the working channel are both acceptable.
- **Video/Pictures tutorial:**
  - Web-based Simulator from the University of Toronto. <http://www.anesthesia.utoronto.ca/edu/cme/bronch.htm>

**3) CAUTIONS**

- **Contraindications:** Relative contraindications
  - Patients at risk of pulmonary and cardiovascular decompensation including patients with severe hypoxemia, hemodynamic instability, current or recent MI
  - Patients at high risk of bleeding
- **Safety Measures:**
  - For the operator: Use of full barrier precautions: cap, mask, gloves and gown. If there is a risk of exposure to airborne diseases, consider deferring the procedure. If necessary personal protective equipment (N95 respirator or PAPR) could be used.
  - For the patient: Patient should not have anything to eat or drink six hr prior to the procedure.
- **Complications:** Bronchospasm, hypoxemia, bleeding, and post procedure fever/sepsis are possible.

**4) ORDER ENTRY (COMMONLY USED SPECIFICATIONS)**

- Bronchial lavage
- Bronchial washing
- Endobronchial biopsy
- Needle aspiration biopsy
- Brushing of endobronchial lesion

**6) REFERENCES**

-Kupeli E, Karnac D, Mehta AC. Flexible bronchoscopy. In: Mason RJ, Broaddus VC, Martin TR, et al., eds. *Textbook of Respiratory Medicine.* 5th ed. Philadelphia, Pa: Elsevier Saunders; 2010:chap 22.

-[Kennedy CC](http://www.ncbi.nlm.nih.gov/pubmed/?term=Kennedy%20CC%5BAuthor%5D&cauthor=true&cauthor_uid=23370487), [Maldonado F](http://www.ncbi.nlm.nih.gov/pubmed/?term=Maldonado%20F%5BAuthor%5D&cauthor=true&cauthor_uid=23370487), [Cook DA](http://www.ncbi.nlm.nih.gov/pubmed/?term=Cook%20DA%5BAuthor%5D&cauthor=true&cauthor_uid=23370487).Simulation-based bronchoscopy training: systematic review and meta-analysis. [*Chest*.](http://www.ncbi.nlm.nih.gov/pubmed/23370487) 2013 Jul;144(1):183-92.
