# Supplementary figures and images for: Checklist for Early Recognition and Treatment of Acute Illness (CERTAIN): evolution of a content management system for point-of-care clinical decision support
Source: BMC Med Inform Decis Mak. 2016 Oct 3;16:127. doi: 10.1186/s12911-016-0367-3 (PMC5048402; doi:10.1186/s12911-016-0367-3)

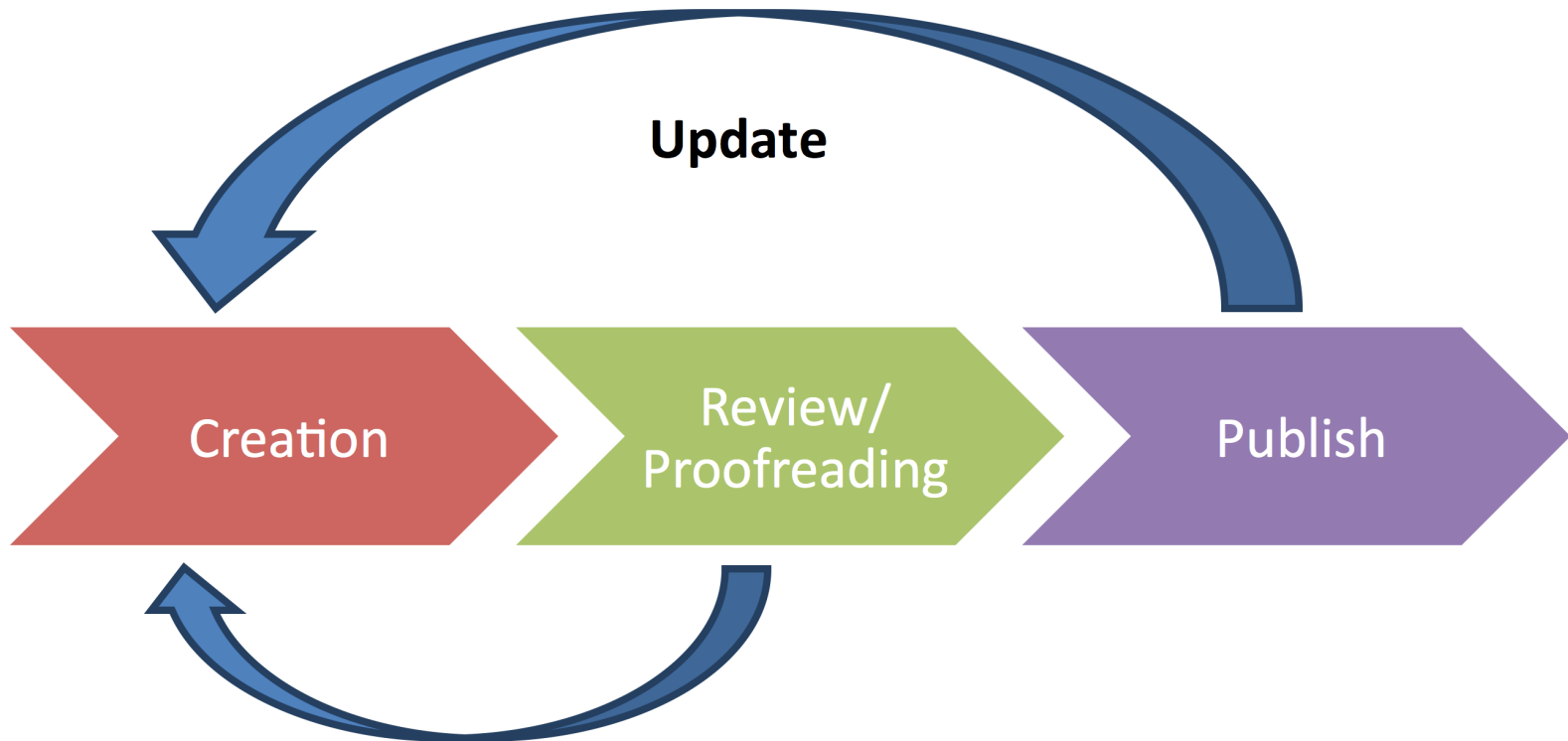

Supplement: Additional file 4: Figure S1. — Card Production Process. After cards are created they undergo iterative cycles of review and content modification. Once finalized and proofed, they are published across the different CERTAIN platforms. Updates, starting the process essentially from the beginning, are performed annually and on an as needed basis. (PDF 181 kb) [file 12911_2016_367_MOESM4_ESM.pdf]

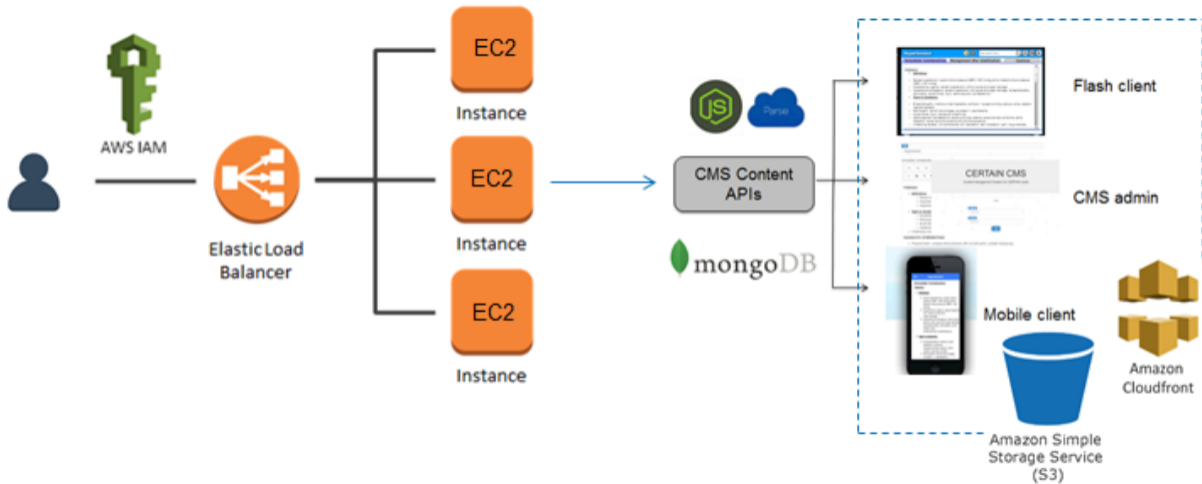

Supplement: Additional file 7: Figure S3. — Overview of customized content management system (for details see Table 2). (PDF 68 kb) [file 12911_2016_367_MOESM7_ESM.pdf]
