# Supplementary material for: Checklist for Early Recognition and Treatment of Acute Illness (CERTAIN): evolution of a content management system for point-of-care clinical decision support
Source: BMC Med Inform Decis Mak. 2016 Oct 3;16:127. doi: 10.1186/s12911-016-0367-3 (PMC5048402; doi:10.1186/s12911-016-0367-3)
Supplement: Additional file 6: — Translation Process. (DOCX 424 kb) [file 12911_2016_367_MOESM6_ESM.docx]

**E-APPENDIX 4.** CERTAIN Content Translation SOP

The main goal of these cards is to provide point of care key-information during the evaluation and treatment of acutely sick patients to ensure that all immediately necessary considerations and actions are done. One of the challenges that we are facing is to make available CERTAIN content to providers which primarily language is other than English. CERTAIN coordination team is putting together an initiative for translating the content material display on the software. To make the translation possible, we are planning to share the content of the cards through Google Drive. <https://drive.google.com/#my-drive>

To have access to CERTAIN please follow the next steps to card to which you were given access:

| 1. Open your personal Gmail account 2. Click on google drive icon | 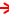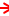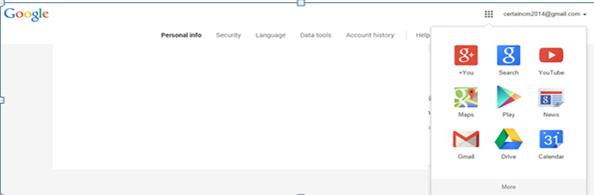 |
| --- | --- |
| 1. Please click on SHARE WITH ME 2. Click on the folder that was shared with you | 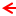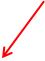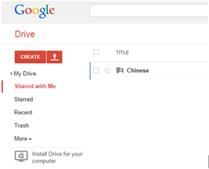 |
| 1. Please click on the card that you will translate as in the pic shows. You have  access to edit | 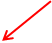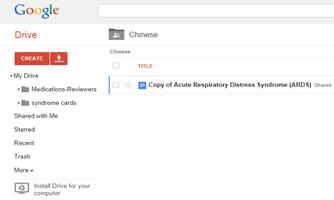 |

**Translating CERTAIN content**

To translate the cards please follow the next steps

1. Create a word document,

| **Creating a Word document** | |
| --- | --- |
| 1. You can download the card doing click on: first file, then download as, finally click on Microsoft word (doc). To open the word document just click on the new doc that shows up on the right lower corner of the screen as in the pic. | 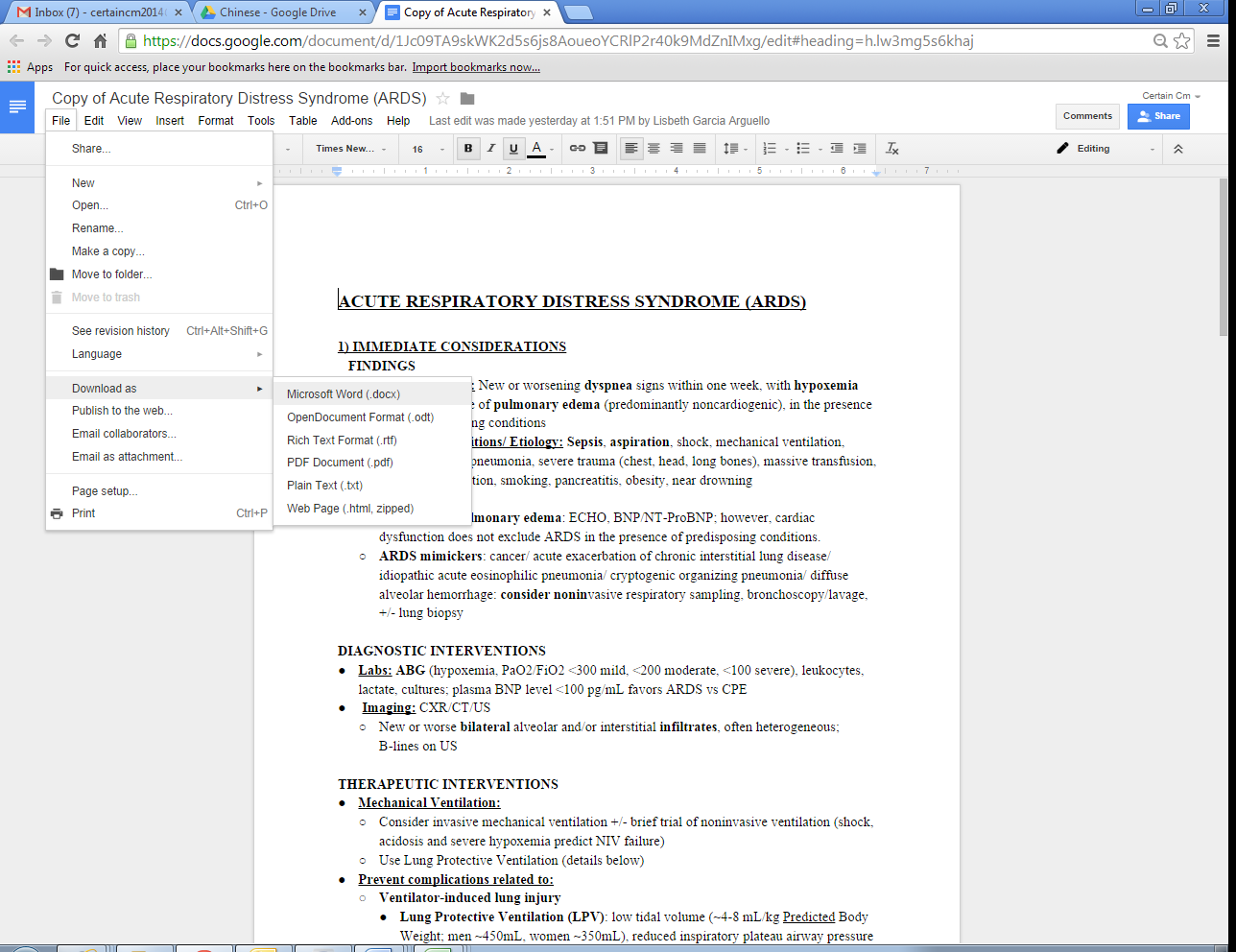  Click file, Download as, Microsoft word  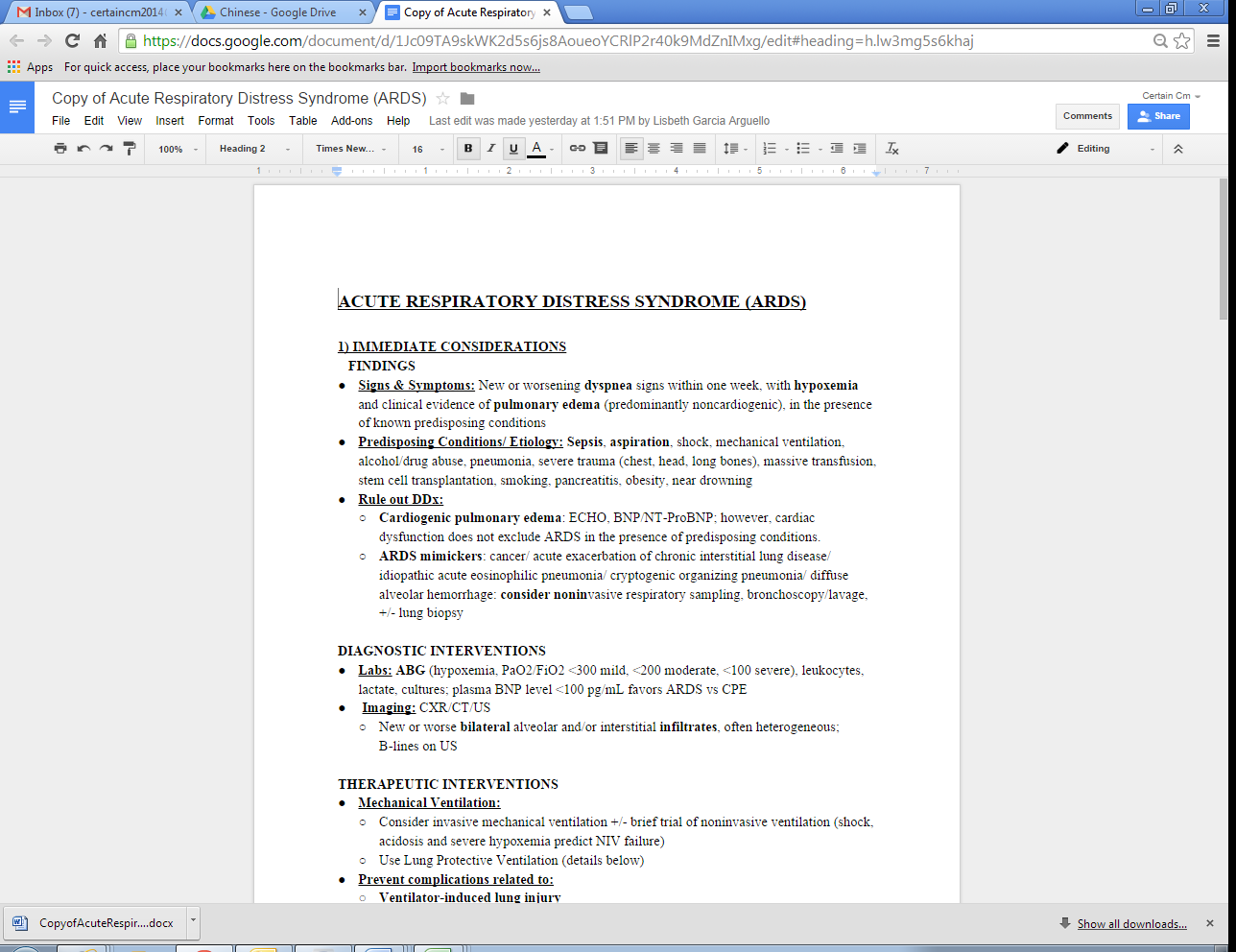  Click |
| 2. Create a world document using the template that is in Appendix 1 (at the end of this SOP) and start your translation by using the real content of the target card. | |
| 3. Once you have finished translating a card, please send it via e-mail to at least to 2 senior faculties (assistant professor or above) at your institution to review it. **Please make sure that anything new added by them (reviewers) needs to be track change mode**. Once you get the card back from the reviewer, review and edit it. | |
| 4. The syndrome card’s name must be written on both language English and on your own language, example XXX (Adult respiratory distress syndrome) | |
| 5. Please upload the final translated card to Google drive and put it on the folder that we are sharing | |
