# Supplementary material for: Checklist for Early Recognition and Treatment of Acute Illness (CERTAIN): evolution of a content management system for point-of-care clinical decision support
Source: BMC Med Inform Decis Mak. 2016 Oct 3;16:127. doi: 10.1186/s12911-016-0367-3 (PMC5048402; doi:10.1186/s12911-016-0367-3)
Supplement: Additional file 5: Figure S2. — Peer-review work flow. For creation of a new card, card expiration and review, or user comments via CERTAIN’s feedback function, a peer review process is initiated. Most issues are resolved on discussion with the author, the content lead editor and the reviewers. However, for complex issues the content management panel can be involved, and if still unclear, final arbitration can be obtained using a modified Delphi process involving the entire expert panel. (PDF 147 kb) [file 12911_2016_367_MOESM5_ESM.pdf]

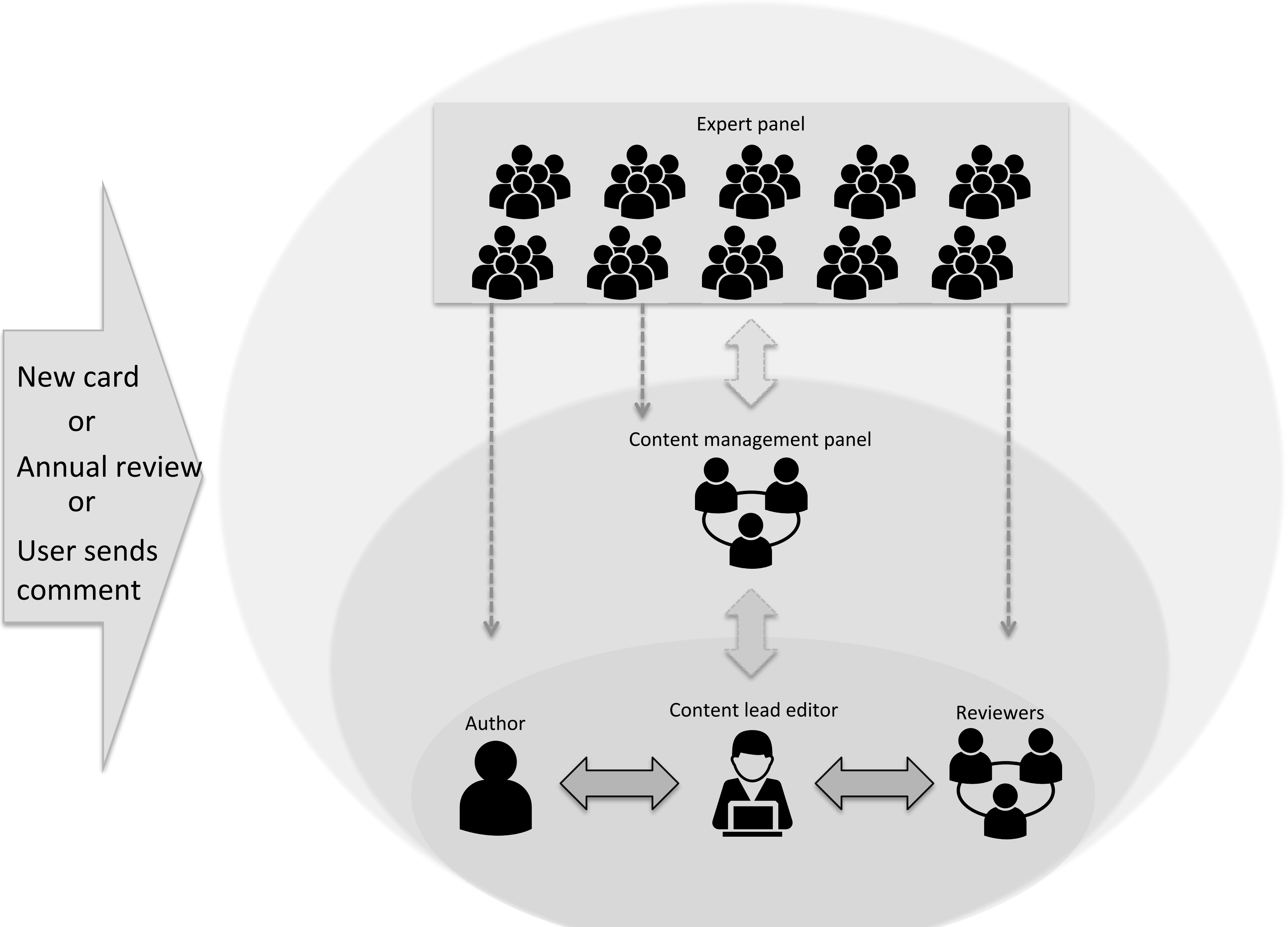

Icons designed by Freepik from www.flaticon.com
